# Effects of vaccination, new SARS-CoV-2 variants and reinfections on post-COVID-19 complications

**DOI:** 10.3389/fpubh.2022.903568

**Published:** 2022-07-29

**Authors:** Mária Orendáčová, Eugen Kvašňák

**Affiliations:** Department of Medical Biophysics and Medical Informatics, Third Faculty of Medicine, Charles University in Prague, Prague, Czechia

**Keywords:** mechanisms, prevention strategies, new research directions, vaccination status, SARS-CoV-2 new variants, post-COVID-19 complications, SARS-CoV-2 reinfections

## Abstract

Post-COVID-19 complications involve a variety of long-lasting health complications emerging in various body systems. Since the prevalence of post-COVID-19 complications ranges from 8–47% in COVID-19 survivors, it represents a formidable challenge to COVID-19 survivors and the health care system. Post-COVID-19 complications have already been studied in the connection to risk factors linked to their higher probability of occurrence and higher severity, potential mechanisms underlying the pathogenesis of post-COVID-19 complications, and their functional and structural correlates. Vaccination status has been recently revealed to represent efficient prevention from long-term and severe post-COVID-19 complications. However, the exact mechanisms responsible for vaccine-induced protection against severe and long-lasting post-COVID-19 complications remain elusive. Also, to the best of our knowledge, the effects of new SARS-CoV-2 variants and SARS-CoV-2 reinfections on post-COVID-19 complications and their underlying pathogenesis remain to be investigated. This hypothesis article will be dedicated to the potential effects of vaccination status, SARS-CoV-2 reinfections, and new SARS-CoV-2 variants on post-COVID-19 complications and their underlying mechanisms Also, potential prevention strategies against post-COVID complications will be discussed.

## Introduction

Long-lasting post-COVID-19 complications emerging in various body systems are reported to have a prevalence of from 8–47.5% in COVID-19 survivors (1–4). Post-COVID-19 complications, also titled as long COVID-19 or post-COVID-19 syndrome (5, 6) are defined as complications occurring or persisting for at least 3 months after an acute SARS-CoV-2 infection and are not attributable to any other etiology other than a SARS-CoV-2 infection (2). There has been intense research done focusing on studying post-COVID-19 symptoms and their relation to structural and functional changes in tissues affected by COVID-19 (7, 8). Some potential mechanisms responsible for the pathogenesis of post-COVID-19 problems have been already investigated (9, 10). Also, it was discovered there are some factors related to acute COVID-19 which are linked with the higher probability of occurrence of post-COVID-19 complications and their severity. The severity and higher probability of occurrence of long-lasting COVID-19-related disturbances were found to be positively associated with the following factors associated with acute COVID-19: some specific acute symptoms (11, 12), severity (13, 14), and long duration of acute COVID-19 (11) and high viral load (15). Vaccination status was associated with a lower risk of exacerbation of post-COVID-19 complications and with lower severity (16, 17). However, to the best of our knowledge, the question of whether there are similarities and differences between vaccinated and unvaccinated COVID-19 survivors regarding the mechanisms underlying post-COVID complications and their effects on the structural integrity of tissues affected by SARS-CoV-2 has not been investigated yet. Also, the effects of SARS-CoV-2 different variants and reinfection cases on dynamics of long-term COVID-19-related complications need to be addressed. Based on the current high rates of infections and reinfections caused by the Omicron variant, we believe these issues represent important research topics. Our hypothesis article is devoted to considerations of the possible effects of vaccination status, different SARS-CoV-2 variants, and reinfection cases on post-COVID complications. Also, potential prevention strategies against post-COVID complications will be discussed. Based on the current knowledge related to post-COVID-19 complications, vaccines against COVID-19, SARS-CoV-2 reinfections, and new SARS-CoV-2 variants, we believe that the proposed issues are relevant and worth studying.

## Post-COVID-19 complications and vaccination status

Vaccination has been demonstrated to be effective in reducing the risk of severe COVID-19 (18, 19) and viral load (20). Also, vaccination status is associated with a significantly reduced risk of becoming infected or reinfected with SARS-CoV-2 (21). Concerning the post-acute period of COVID-19, vaccines were already investigated as a potential treatment for post-COVID-19 complications but with mixed results. Both improvement and worsening of post-COVID-19 symptoms were observed in post-COVID-19 patients after receiving vaccines (22).

### Post-COVID symptoms in vaccinated and unvaccinated populations

Based on the recent findings, vaccination status seems to represent significant prevention of developing severe and long-term post-COVID-19 complications in adults and children (16, 17). Two doses of vaccine were found to be effective in reducing the risk of exacerbation of Multi-System Inflammatory Syndrome in the post-COVID period (16). Multi-System Inflammatory Syndrome is a hyper-inflammation disorder (23) associated with COVID-19 (24). This particular disorder develops during the sub-acute/post-acute COVID-19 period (23–25). Although Multi-System Inflammatory Syndrome usually occurs in children (23, 24), there is evidence it may occur in adults too (25). Other evidence supporting the effectiveness of vaccination status as protection against post-COVID-19 complications comes from Kuodi et al. (17). The aim of that study was to investigate the severity and duration of post-COVID-19 complications in vaccinated and unvaccinated people who got infected by SARS-CoV-2. Investigated post-COVID-19 complications included fatigue, headaches, limb weakness, myalgia, loss of concentration, hair loss, insomnia, dizziness, persistent cough, and shortness of breath. Based on data obtained from medical questionnaires given to respondents, the duration and severity of post-COVID-19 symptoms were significantly reduced in the vaccinated population compared to the unvaccinated one (17). In addition, vaccination status was also positively associated with a considerably lower risk of exacerbating post-COVID-19 complications (17). Other intriguing findings supporting vaccination status as effective prevention of long-term post-COVID complications come from the prospective cohort study which investigated differences between vaccinated and unvaccinated populations suffering from a post-COVID condition (26). It was discovered that receiving at least one vaccine dose against COVID-19 prior to SARS-CoV-2 infection was significantly positively correlated with lower odds of having respiratory failure, hypoxemia, and oxygen support requirement (26). Post-COVID muscle diseases, anosmia, hypercoagulation, seizures, psychotic diseases, and hair loss were also significantly reduced in the vaccinated population (26). On the other hand, the prevalence of post-COVID anxiety, sleep disorders, mood disorders, and renal diseases did not differ between vaccinated and unvaccinated populations (26). These findings suggest that some mechanisms responsible for the pathogenesis of post-COVID manifestations can be eliminated by vaccination whereas some other mechanisms might come into play regardless of vaccination status.

### Possible differences in mechanisms responsible for post-COVID complications between vaccinated and unvaccinated populations

Although post-COVID-19 symptoms may occur in vaccinated people as well (17, 26, 27), the findings above show the considerable effectiveness of COVID-19 vaccines in reducing the severity and duration of post-COVID-19 complications as well as reducing the risk of occurrence of some particular post-COVID complications. At the same time, these findings give rise to intriguing questions regarding the mechanisms underlying the protective effects of vaccination against post-COVID-19 complications. Vaccination status is associated with a significant reduction in the risk of severe COVID-19 (19, 28). Severe COVID-19 is frequently accompanied by multi-organ dysfunctions (29) which give rise to exacerbations of the pathological processes responsible for post-COVID-19 complications (29, 30). For that reason, we propose vaccination status may be associated with a reduced risk of exacerbation of post-COVID complications stemming from pathologies associated with severe COVID-19.

The prevalence of some post-COVID complications, such as anxiety, sleep disorders, and mood disorders, did not differ between vaccinated and unvaccinated people who recovered from COVID-19 (26). The occurrence of anxiety, mood disorders, and sleep disturbances in both populations was interpreted as a consequence of psychological factors, such as fears of getting SARS-CoV-2 infections despite being vaccinated, which are hardly modifiable by vaccination status (26). However, more future research would be needed to confirm or reject this hypothesis. In relation to post-COVID-19 renal problems, one possible explanation is that the prevalence of renal problems in both vaccinated and unvaccinated COVID-19 survivors might be attributable to age which was found to be positively associated with the occurrence of severe COVID-19-related complications including renal diseases (31). This postulate might be at least partly supported by a positive association between age and lower level of protection by COVID-19 vaccines against acute SARS-CoV-2 infection (28) and post-COVID-19 complications (32, 33). Based on the documented link between alterations in gut microbiota and various neurological problems including sleep and mood disturbances (34), it is also possible that post-COVID sleep problems and mood disturbances are attributable to alterations in gut microbiota. This hypothesis might be supported by pieces of evidence of alterations of gut microbiota in people suffering from post-COVID problems (35, 36). In contrast to a healthy population and COVID-19 survivors with no post-COVID health issues, the diversity of gut microbiota in COVID-19 survivors with the presence of post-COVID manifestations was found to be significantly reduced (35, 36). Furthermore, the presence of post-COVID disturbances was associated with the enrichment of opportunistic pathogens and depletion of beneficial commensals (35, 36). Positive correlations were found between some long-lasting COVID-19-related health problems and changes in gut microbiota. For instance, counts of *Bifidobacterium* and *Faecalibacterium prasnitzi*, whose depletion is associated with anxiety and worsened sleep quality (34), were significantly reduced in people suffering from post-COVID complications (36). A negative correlation was found between counts of *Faecalibacterium prasnitzi* and the severity of post-COVID chest pain (35). Dysfunction of gut microbiota can also cause renal diseases (37–39). Taking into consideration the notion that the prevalence of post-COVID renal diseases, sleep problems, and mood disturbances did not differ between vaccinated and unvaccinated populations (40) and provided that the particular post-COVID complications stemmed from dysfunctions of gut microbiota, it may be assumed that vaccination status is less likely to protect from COVID-related dysfunctions of gut microbiota. However, in order to verify this hypothesis, future studies are needed to investigate relations between gut microbiota changes after COVID-19, the severity of particular post-COVID complications, and vaccination status.

Last but not least, it is necessary to take into consideration external factors which are unrelated to vaccination status and that might be responsible for the same prevalence of sleep disorders, mood disturbances, and renal problems in vaccinated and unvaccinated COVID-19 survivors. For instance, in dialysis patients, there may come extra health complications such as infection by *Staphylococcus aureus* from peritoneal catheter exit-site (41) which may lead to peritonitis that may further worsen and complicate the health condition of patients suffering from renal problems (42). In COVID-19 survivors, sleep problems, and mood disturbances may also exacerbate secondarily as a consequence of renal problems and/or other present health complications.

In contrast to post-COVID anxiety and mood disorders, vaccination status was associated with a considerably reduced prevalence of post-COVID neurological manifestations such as seizures, psychotic disorders, and anosmia (26). Based on these findings, it is possible that vaccination status eliminates some mechanisms responsible for the exacerbation of these kinds of neurological manifestations. For instance, elevated levels of some pro-inflammatory cytokines were found to be capable of eliciting seizures by lowering the seizure threshold in neuronal populations (43). Excessive levels of pro-inflammatory cytokines are attributed to maladaptive immune processes during acute (32) and/or post-acute COVID-19 (33). Based on this notion, it is possible that vaccination status may somehow prevent COVID-19-related exaggerated or otherwise maladaptive immune responses to SARS-CoV-2 infection. This proposal might be at least partially supported by a positive association between vaccination status and significantly reduced occurrence of post-COVID hypercoagulopathy condition (26), as hypercoagulopathy can be associated with maladaptive immune processes in COVID-19 (44, 45). Another supportive argument for the possible link between decreased probability of COVID-related exaggerated immune processes and post-COVID complications stemming from COVID-related exaggerated immune processes might be found in significantly reduced odds of developing Multi-System Inflammatory Syndrome after COVID-19 in the vaccinated population (16). Vaccination status was also associated with a significantly reduced prevalence of post-COVID anosmia, respiratory failure, and muscle disease /neuromuscular junction disease (26). Since these listed post-COVID complications represent multi-organ involvement, it is possible that vaccination status represents significant prevention from post-COVID complications originating from multi-organ COVID-related pathology and lung-dependent hypoxia associated with severe forms of COVID-19 requiring hospitalizations. These postulates might be at least partially supported by the positive correlation between vaccination status and a significantly reduced rate of prevalence of severe COVID-19 (16, 18, 26, 28). Multi-organ COVID-19-related pathology which is frequently associated with severe COVID-19 (46) is attributed to direct invasion of SARS-CoV-2 *via* ACE2 receptors, Neuropilin 1, and Transmembrane serine 2 proteases (TMPRSS2) (47–49), COVID-19-related cytokine storm and COVID-related hypoxia (9, 50). Since vaccines against COVID-19 are effective at reducing viral loads (20, 51), it is possible that vaccination status might prevent COVID-19-related multi-organ pathology by preventing excessive virus dissemination within organisms *via* reducing viral loads and improving immune functioning.

However, it is necessary to mention our considerations about the possible effects of vaccines on the elimination of the aforementioned proposed mechanisms responsible for post-COVID conditions are limited due to the lack of studies investigating this issue. Therefore, future research is needed to clarify whether vaccination status eliminates mechanisms responsible for post-COVID symptoms originating from maladaptive immune processes, lung-dependent hypoxia, and multi-organ pathologies.

Apart from COVID-19-related pathology stemming from direct effects of SARS-CoV-2, vaccination status might represent significant prevention from post-COVID symptoms caused by secondary pathological processes related to SARS-CoV-2 infection. For instance, the prevalence of post-COVID posttraumatic stress disorder PTSD (50), which can be triggered by hospital stay in intensive care units due to the fears of death (52), might be likely decreased by vaccination status due to vaccine-related protection of severe COVID-19 requiring hospitalization. Also, the risk of COVID-related muscle atrophy caused by immobility in being hospitalized in intensive care units might be eliminated due to vaccine-related prevention from severe COVID-19 forms requiring hospitalization (26, 28). To sum it up, vaccination status might represent good prevention of developing post-COVID complications triggered by secondary pathological processes associated with severe COVID-19 requiring hospitalizations.

The following Table 1 gives an overview of our considerations about mechanisms responsible for post-COVID complications which might be possibly reduced by vaccination.

**Table 1:** Mechanisms responsible for post-COVID complications which might be possibly reduced by vaccination.

**Mechanisms responsible for post-COVID complications which might be possibly reduced by vaccination**
**1**. Lung-dependent hypoxia
**2**. Exaggerated immune responses to SARS-CoV-2 infection
**3**. COVID-related multi-organ failure/dysfunction
**4**. Post-COVID complications such as muscle atrophy and PTSD attributable to indirect effects of COVID-19-related hospitalizations

There is documented link between vaccination status and reduced severity, duration, number, and some particular types of post-COVID complications (16, 17, 26). In addition, vaccination status was found to be associated with reduced odds of some acute manifestations of COVID-19 such as its severity (18, 26, 28), the simultaneous occurrence of more than 5 acute symptoms (28), and their durations (28), anosmia (28). All these factors are associated with a higher risk of developing post-acute COVID-related manifestations (11–13, 50, 53, 54). Therefore, documented vaccine-induced elimination of these factors might be causally connected with mechanisms related to vaccine-induced prevention of post-COVID complications.

The following Table 2 gives a list of vaccine-related reductions of acute COVID-19 symptoms associated with a higher risk of post-COVID complications.

**Table 2:** List of vaccine-related reduction of acute COVID-19 manifestations associated with a higher risk of post-COVID complications.

**Acute COVID-19 manifestations are associated with higher risk of post-COVID symptoms whose prevalence was found to be reduced in vaccinated people infected by SARS-CoV-2 variants**
**1**. severe form of COVID-19 requiring hospitalization
**2**. Long duration of clinical acute COVID-19 symptoms
**3**. Simultaneous occurrence of more than 5 acute COVID-19 symptoms
**4**. Prevalence of anosmia
**5**. High viral load

However, there are some limitations in our consideration that need to be mentioned. First, it is not known how the listed acute manifestations influence mechanisms responsible for post-COVID complications. Second, it is not known whether the documented link between particular acute COVID-19 symptoms and risk of developing post-acute COVID-related manifestations is universal, or it depends on the factors such as vaccination status and infections by different SARS-CoV-2 variants. Third, it remains elusive whether vaccine-induced reduction of the prevalence of aforementioned acute COVID-19 symptoms might prevent mechanisms responsible for structural and functional abnormalities of tissues and organs related to post-COVID manifestations.

### The possible effect of vaccination status on structural correlates of post-COVID manifestations

Post-COVID manifestations frequently go hand in hand with structural damage to organs and tissues caused by COVID-19 (30, 55, 56). The severity of post-COVID complications was repeatedly documented to correlate positively with the severity of structural and metabolic abnormalities of tissues (8, 57–60). For example, the severity of some neurological post-COVID complications was found to be associated with the level of COVID-related structural damage to neuronal tissue and brain metabolic abnormalities (8, 55, 57, 58, 60). Also, the severity of pulmonary post-COVID complications was found to correlate directly with the levels of reduction in diffusion capacity in post-COVID conditions (59). Since vaccination status was found to be associated with reduced duration of post-COVID-19 symptoms and their decreased severity (17, 26), it is, therefore, possible that COVID-related tissue damage will be significantly less pronounced in vaccinated COVID-19 survivors suffering from post-COVID disturbances compared to the unvaccinated population. Our proposal might be at least partly supported by the fact that vaccination status significantly reduced the occurrence of severe forms of COVID-19 requiring hospitalizations (26, 28). Since post-COVID clinical and radiological picture may display characteristics of post-intensive care syndrome (50) in which structural and functional abnormalities related to multi-organ dysfunctions might occur (61, 62), it is possible that these conditions related to COVID-19 pathology and their underlying structural and metabolic correlates would be reduced in vaccinated population after SARS-CoV-2 infection.

In addition, vaccination status might indirectly prevent structural abnormalities attributable to secondary processes induced by COVID-related pathology. For instance, due to the absence of physical activity, muscle atrophy can be present in COVID-19 survivors after their hospitalization in intensive care units (63). We believe that future research will be needed to disentangle possible differences in the effects of post-COVID manifestations on tissue integrity between vaccinated and unvaccinated populations.

## Post-COVID-19 complications vs. SARS-CoV-2 reinfections

Because the world is currently facing the highly contagious SARS-CoV-2 Omicron variant, which is causing a high rate of new infections and reinfections (64), we believe that the link between post-COVID-19 complications and SARS-CoV-2 reinfections is worth-studying. Reinfection with SARS-CoV-2 is attributed to several factors such as the time-dependent decline in antibodies gained from the first infection or vaccination, underlying immunological comorbidities, and the occurrence of new SARS-CoV-2 strains (19). Reinfections with SARS-CoV-2 were frequently associated with milder forms of COVID-19 (65) compared to the severity of COVID-19 associated with the initial infection. However, there is also evidence that reinfections with SARS-CoV-2 may be associated with an even worse form of acute COVID-19 than during the initial infection (66). Since the severity of acute COVID-19 is positively associated with a higher risk of long-lasting post-COVID-19 complications (13, 67), we believe that not only initial SARS-CoV-2 infections but also reinfections might be followed by post-COVID-19 complications. To the best of our knowledge, the effect of SARS-CoV-2 reinfections on post-COVID complications has not been systematically studied yet. Nevertheless, two recent case studies describe the occurrence of post-COVID complications following SARS-CoV-2 reinfections.

A case study done by Penetra et al. (27) demonstrated that SARS-CoV-2 reinfection could lead to post-COVID-19 complications. This case study described the case of a COVID patient who was initially infected by B.1.1.3.3. SARS-CoV-2 variant. After the initial SARS-CoV-2 infections, no post-COVID manifestations occurred. However, after more than 1 year after the initial SARS-CoV-2 infection, there came reinfection with the Gamma SARS-CoV-2 variant. Reinfection was followed by post-COVID headaches and blurred vision (27). Another case study revealed completely different dynamics of post-COVID condition following SARS-CoV-2 reinfection (68). In this case study, the patient experienced psychotic post-COVID symptoms lasting for several months which followed the initial SARS-CoV-2 infection. Prior to SARS-CoV-2 reinfection, the patient stated complete recovery of her condition. However, after SARS-CoV-2 reinfection, there came to exacerbation of very similar psychotic manifestations as was in the case after the initial infection (68).

It can be seen that post-COVID complications following SARS-CoV-2 reinfection may occur in people who did not develop any post-COVID complications after initial infection (27) as well as in people who have positive anamnesis of post-COVID complications after initial infection (68). So far, it remains unknown which putative mechanisms might come into play to cause post-COVID complications after SARS-CoV-2 reinfection.

### Possible mechanisms responsible for post-COVID manifestations following SARS-CoV-2 reinfections

It is possible that the particular post-COVID problems following the second SARS-CoV-2 infection could be triggered by reactivation of the previous post-COVID problems following the initial SARS-CoV-2 infection. An alternative explanation is that COVID-related disturbances following SARS-CoV-2 reinfection may be independent or at least partially independent of post-COVID condition following the initial SARS-CoV-2 infection. The first possibility speaking in favor of reinfection-related reactivation of previous post-COVID problems related to the first SARS-CoV-2 infection might be at least partially supported by the aforementioned case of a psychotic patient in YarlaGadda et al. (68) study who experienced a similar psychotic problem after initial SARS-CoV-2 infection as well as after the second SARS-CoV-2 infection. Another supportive argument for this hypothesis might come from studies discovering positive correlations between the positive anamnesis of neuropsychiatric conditions and higher odds of developing post-acute COVID-related manifestations (50, 69). The second possibility points toward total or at least partial independence between post-COVID complications following initial SARS-CoV-2 infection and post-COVID complications following SARS-CoV-2 reinfection. This postulate can be at least partly supported by pieces of evidence of cases of COVID-19 survivors suffering from post-COVID complications who managed to completely recover from their conditions (70, 71). However, apart from the aforementioned two case studies, the link between SARS-CoV-2 reinfections and post-COVID complications has not been studied yet. For that reason, our hypotheses need to be further investigated by future research.

Considering the high rate of new infections and reinfections (64, 72), we believe that studying the potential similarities and differences in the occurrence of post-COVID-19 symptoms between the post-acute period after initial SARS-CoV-2 infection and the post-acute period after SARS- reinfection seem to be worth-studying. Future studies should also investigate whether particular post-COVID-19 complications are more likely to occur after initial SARS-CoV-2 infection or after reinfection.

### Immune profile of COVID-19 survivors

It remains unknown which factors are associated with a higher risk of developing post-COVID complications after SARS-CoV-2 reinfection. It is also unclear whether there are some differences in the probability of developing post-COVID complications after SARS-CoV-2 reinfection between those who developed and those who did not develop any post-COVID complications after initial SARS-CoV-2 infection.

Some COVID-19 survivors were found to exhibit high levels of humoral and cellular-specific immune markers against SARS-CoV-2 (73). On the contrary, there are people who were found to exhibit low (73) or even no levels of the particular immunologic markers (74). For that reason, it might be assumed that people with low levels of specific immunity targeted against COVID-19 will be at higher risk of post-COVID complications simply due to the higher risk of getting reinfected with SARS-CoV-2. Also, people with deregulated immune processes might be at a higher risk of developing post-COVID complications after SARS-CoV-2 reinfection. In the acute and/or post-acute phase of SARS-CoV-2 infection, there may come deregulation of immune processes (7, 33, 75) due to COVID-19-related pathological processes such as cytokine storms (32, 66), hypercoagulopathy, dysautonomia (76) and the emergence of autoantibodies (77). Post-COVID-19 complications can originate from exaggerated and/or otherwise abnormal immune responses during acute SARS-CoV-2 infection (7, 78). For that reason, people suffering from deregulations of their immune system caused by initial SARS-CoV-2 infection may be at risk of developing post-COVID-19 following SARS-CoV-2 reinfection. In addition, people with long-term immune deregulation after initial SARS-CoV-2 infections might be theoretically at higher risk of developing immune-mediated post-COVID complications after SARS-CoV-2 reinfection because of decreased abilities of their immune system to combat pathogens. Furthermore, a weakened or otherwise deregulated immune system might also represent a risk of getting infected by other pathogens than SARS-CoV-2 which might represent a risk condition for exacerbation of post-COVID problems. This postulate might be supported by the documented positive correlation between the acute presence of other infections parallel to SARS-CoV-2 and higher odds of developing long-term post-COVID complications (79).

Some immune markers associated with immune deregulation, such as elevated levels of pro-inflammatory cytokines, presence of autoantibodies, and abnormal distribution of leukocytes following acute COVID-19 were positively correlated with the presence and severity of various post-COVID complications (33, 79, 80). Therefore, it is possible, that people who have post-COVID-19 complications stemming from deregulated immune systems might be more likely to worsen their condition after reinfection with SARS-CoV-2 compared to people with no positive anamnesis of post-COVID manifestations. This postulate might be at least partly supported by several studies in which significant differences were found in immunological profiles between COVID-19 survivors with a positive anamnesis of post-COVID complications and COVID-19 survivors with no post-COVID complications (7, 13, 81).

## Post-COVID-19 vs. SARS-CoV-2 different variants

The notion of the possibility of reinfection by SARS-CoV-2 variants gives rise to questions regarding emerging new SARS-CoV-2 variants. The emergence of new SARS-CoV-2 variants represents a formidable challenge for health care systems as new variants may escape the immune responses developed after initial infections and/or after vaccination, which appears to be the case for Omicron variants (82, 83). So far, major variants of concern include the Alpha, Beta, Gamma, Delta, and Omicron SARS-CoV-2 variants (84, 85). These strains were found to differ in several aspects such as incubation period (86, 87), transmissibility (83), the severity of COVID-19 (88–90), viral load (91), acute symptoms of COVID-19 (88, 92), degree of vaccine resistance (19, 82, 83), degree of natural immunity gained after recovery from previous SARS-CoV-2 infections (72), and age group vulnerability (92).

The probability of developing post-COVID-19 complications was positively associated with factors such as exposure to high viral loads (15), the severity (13, 67), and duration (11, 14) of acute COVID-19 (13, 67) as well as a number of clinical symptoms co-occurring during acute COVID-19 (13). Also, being unvaccinated is associated with a higher risk of developing post-COVID manifestations (16, 17). In addition, some COVID-19 symptoms such as fatigue (11, 54), chest pain (12), fever (11, 12), diarrhea, headaches (12, 54), dyspnea (54, 67), and olfactory impairment (12, 54) were associated with higher risks of developing long-term COVID-19- related health complications.

Existing SARS-CoV-2 variants of concern were found to differ in some of the factors mentioned above. For example, differences in viral load (91), the severity, and specific symptoms of acute COVID-19 (88, 92) were found between SARS-CoV-2 variants.

Furthermore, SARS-CoV-2 variants seem to differ from each other in the age populations vulnerable to the particular SARS-CoV-2 variant. In contrast to the previous SARS-CoV-2 variants of concern affecting predominantly elderly populations (93), the younger population seems to be a predominant target of the Omicron SARS-CoV-2 variant (94). Consequently, it might be of great importance to investigate whether there might be differences in post-COVID complications and their underlying mechanisms between the particular SARS-CoV-2 variants.

So far, there were several recent studies done to investigate possible differences between post-COVID complications caused by different SARS-CoV-2 variants (95–97). The prospective cohort study revealed no significant difference in the prevalence of the post-COVID complications such as fatigue, shortness of breath, musculoskeletal pain, cough, heart palpitations, anxiety, and depression between the population infected with Omicron and the population infected with Delta variants (96). These findings seem to be in line with other studies in which no significant differences were found in the prevalence of post-COVID fatigue, shortness of breath, hair loss, depressed mood, brain fog, loss of concentration, and memory disturbance between post-COVID conditions caused by Omicron and Delta SARS-CoV-2 infections (97). It can be seen that investigated types of post-COVID symptoms from studies (96) and (97) hugely overlap. Based on these outcomes, there seems to be no significant difference between types of post-COVID complications stemming from infections caused by Omicron and Delta variants. Another common feature between Omicron-related and Delta-related post-COVID conditions was found in the time-dependent reduction of risk of exacerbation of health complications after COVID-19 (96). However, there were some differences found in the prevalence of post-COVID complications as such (95, 96). It was discovered that the prevalence of post-COVID fatigue and shortness of breath following 3 months after SARS-CoV-2 infection is significantly higher in people who were infected with Delta compared to those who were infected with Omicron (96). This outcome is at least partly consistent with another study which demonstrated the significantly greater prevalence of post-COVID complications in the pandemic wave in which the Delta variant dominated (June-November 2021) compared to the pandemic wave with the greatest predominance of the Omicron variant (December 2021-March 2022) (95). In the same vein, one cross-sectional study found a lower probability of occurrence of long-term COVID-related disturbances following Omicron infection compared to the prevalence of post-COVID complications stemming from SARS-CoV- 2 infections from previous pandemic waves (97). According to consistency in these studies, there seems to be a lower probability of developing long-term health complications following Omicron infections than in the case of the previous variants of concerns. Since previous variants of concern such as alpha and delta were linked with a significantly higher rate of severe COVID-19 forms requiring hospitalizations than Omicron (83), there might be the same trend for the evolution of dynamics of post-COVID complications. In other words, it is possible evolution of post-COVID symptoms following Omicron and future SARS-CoV-2 variants will lead to lower prevalence as well as lower severity and duration of the particular post-COVID health problems. Such a scenario would be in accordance with the evolutionary perspective of a gradual process of adaptations of the virus on the host to evolve to a more contagious but less pathogenic variant (84). However, there are some limitations in our considerations that need to be mentioned. First, there is a gap of knowledge regarding differences in the level of severity of post-COVID complications caused by Omicron and other SARS-CoV-2 variants. The aforementioned discussed studies investigating differences in types between post-COVID problems caused by Omicron and Delta variants focused only on studying the particular types of the particular health complications but not on their severity (95–97). For that reason, we propose questionnaires, scales, and inventories used for assessing the severity of medical symptoms should be used. Secondly, a combination of longitudinal and multi-center cross-sectional studies should be used to monitor the relation between post-COVID complications and particular SARS-CoV- 2 variants in order to discover long-term dynamics of post-COVID conditions across the populations differing in their demographic characteristics. This may be relevant to the fact that factors such as gender and age were found to play important role in determining the vulnerability to different SARS-CoV-2 variants (93, 94, 96). A more detailed view of our proposed strategies for studying the effects of SARS-CoV-2 variants on post-COVID complications is presented in chapter 4.2 Possible strategies for investigating effects of vaccination status, SARS-CoV-2 new variants, and reinfections on post-COVID-19 complications. Also, the question of whether there are differences in underlying mechanisms of pathogenesis of the particular post-COVID condition caused by various SARS-CoV-2 variants might be worth-studying.

### Mechanisms responsible for the exacerbation of post-COVID complications might differ with regard to the SARS-CoV-2 variant

Studies focusing on the Omicron SARS-CoV-2 variant found that anosmia, which is associated with a higher probability of post-COVID-19 complications (12, 54), is less common in Omicron compared to other SARS-CoV-2 variants (89, 98–100). Therefore, if Omicron causes neurological post-COVID-19 complications, these complications might be less likely to stem from damage to olfactory tissues and adjacent brain areas caused by direct entry of SARS-CoV-2 to the olfactory system. Post-COVID-19-related anosmia has been associated with damaged tissue in the olfactory neuroepithelium (101) and the presence of SARS-CoV-2 in the olfactory neuroepithelium (101) and hypometabolism in brain areas involved in the olfactory pathways in COVID-19 survivors (8, 60). Provided that Omicron does not attack the olfactory epithelium, it is possible that post-COVID complications caused by Omicron are less likely to be accompanied by anosmia and structural and functional abnormalities in the olfactory system as it were in the case of the previous SARS-CoV-2 strains. This postulate might be at least partly supported by differences in mechanisms regarding cell entries between some SARS-CoV-2 variants. Compared to previous SARS-CoV-2 variants such as Delta, Omicron seems not to use transmembrane protease serine 2 (TMPRS2) for cell entry (49). Since TMPRS, Neuropilin-1, and ACE2 are essential for neuroinvasion by SARS-CoV-2 in non-neural cells and olfactory epithelia (102), it is possible that Omicron might be less effective in invading olfactory epithelia due to its inability to enter cells *via* TMPRS thereby reducing the probability of exacerbation of post-COVID complications related to olfactory impairment. However, thorough molecular and clinical research would be needed to confirm or reject this hypothesis.

Contrary to previous SARS-CoV-2 variants of concern, based on the symptomatology of patients infected by Omicron, preliminary findings suggest symptoms of acute SARS-CoV-2 infection seem to be predominantly related to the upper respiratory tract (100, 103) in which Omicron variant was demonstrated to replicate more rapidly compared to lower respiratory pathways (104, 105). Also, the Omicron SARS-CoV-2 infection is documented to be associated with reduced rates of hospitalizations requiring oxygen support (92, 94, 106). Based on these characteristics of the Omicron variant, it is possible that post-COVID complications caused by Omicron are less likely to originate from lung-dependent hypoxia which was the more common case in previous pandemic waves (107). Also, indirect effects of hospitalization such as muscle atrophy (63) and PTSD (52), might be less likely to develop in Omicron-related post-COVID manifestations. This postulate might find its support in characteristics of Omicron infection. In contrast to previous SARS-CoV-2 variants, Omicron infections requiring hospitalizations are characterized by decreased disease severity and shorter stay in the hospital (92, 94, 106). In addition, both disease severity and the long duration of acute COVID-19 are associated with higher odds of developing post-COVID manifestations (11–13, 50). These outcomes possibly speak in favor of a lower probability of developing post-COVID symptoms following SARS-CoV-2 infection by Omicron.

Concerning Omicron, there might be factors associated with a higher risk of developing post-COVID-19 complications such as reduced vaccine effectiveness against Omicron (83) and specific acute symptoms including headaches, fatigue, and fever (92, 108, 109). In pandemic waves prior to Omicron, the presence of these specific acute symptoms was associated with a higher risk of exacerbation of post-COVID-19 complications (11, 12). It remains to be investigated whether these factors will be also associated with a higher risk of post-COVID-19 complications following Omicron SARS-CoV-2 infections.

The following Table 3 summarizes our considerations about the factors indicating low probability and factors indicating a high probability of exacerbation of post-COVID complications after infection with Omicron.

**Table 3:** Factors indicating a low and high probability of occurrence of post-COVID-19 complications caused by SARS-CoV-2 Omicron infection.

**Factors indicating high probability of occurrence of post-COVID-19 complications caused by Omicron infection**	**Factors indicating low probability of occurrence of post-COVID-19 complications caused by Omicron infection**
**1**. Omicron significantly evades immunity gained from vaccination and past Sars-CoV-2 infectons.	**1**. In case of Omicron infections, there is reduced rate of severe COVID-19 forms requiring hospitalizations.
**2**. Symptoms such as fatigue, headaches and fever, that are associated with higher risk of exacerbation of post-COVID-19 complications, seem to be frequent in Omicron Sars-CoV-2 infections.	**2**. In case of Omicron infections, there is reduced prevalence of anosmia (risk factor for the occurrence of post-COVID-19 complications).

However, it is necessary to mention some limitations in our considerations. First, while there are studies reporting lower rates of occurrence of hypo/anosmia in SARS-CoV-2 infections caused by Omicron (89, 98, 99), there are also reports showing no significant differences in hypo/anosmia between SARS-CoV-2 infections caused by Omicron vs. other SARS-CoV-2 variants (110).

Second, it is not clear whether Omicron causes considerably lower numbers of severe COVID-19 cases than other variants, and even provided that it does, it is unclear whether the reportedly milder infections caused by Omicron is an inherent attribute or whether it can be ascribed to the higher levels of collective immunity due to vaccinations and previous SARS-CoV-2 infections (111). Furthermore, even if the Omicron causes fewer cases of severe COVID-19 than previous strains, its transmissibility is 3.31-fold higher than it was for the Delta SARS-CoV-2 variant (112). Statistically speaking, even though the Omicron variant seems to be associated with lower hospitalization frequency (106), the great transmissibility of this variant may result in more hospitalizations (6) resulting in a high number of COVID-19 survivors suffering from post-COVID-19 complications stemming from severe forms of COVID-19.

Nevertheless, there are also reports speaking in favor of increased rates of hospitalizations linked with Omicron (110, 113). For instance, one study investigating characteristics of Omicron and Delta SARS-CoV-2 variants found Omicron and Delta are associated with comparable viral loads plus those two variants do not differ significantly between rates of severe COVID-19 requiring oxygen support (113). Such similarities in characteristics of these two variants might indicate no significant differences in post-COVID complications caused by these two SARS-CoV-2 variants. However, it is unknown whether the prevalence of post-COVID-19 complications caused by Omicron will be positively correlated with the severity of acute COVID-19.

Furthermore, it is not known whether acute COVID-19 symptoms, which were associated with higher odds of developing post-COVID complications in pandemic waves preceding the Omicron wave, would be associated with a higher probability of developing post-COVID manifestations following Omicron infection.

Considering that Omicron and/or future SARS-CoV-2 variants may be associated with fewer severe COVID-19 hospitalizations requiring oxygen therapy, this could lower the probability of severe post-COVID-19 complications. However, since post-COVID-19 symptoms can occur even after mild or asymptomatic SARS-CoV-2 infection (114, 115), the risk of post-COVID-19 complications following SARS-CoV-2 infection by the Omicron variant or future variants should be not underestimated.

Also, studying whether the severity of acute COVID-19 and the associated acute symptoms caused by a particular SARS-CoV-2 variant correspond to types of post-COVID-19 types and their severity would be interesting and relevant from the evolutionary point of view as well as to the need with developing new treatment and prevention strategies against post-COVID complications.

So far, we discussed our considerations related to the possible effects of the vaccination status, SARS-CoV-2 new variants, and reinfections on post-COVID-19 complications. In the next section, we will discuss how future studies, dedicated to addressing the aforementioned issues, could be carried out.

## Possible strategies for investigating effects of vaccination status, SARS-CoV-2 new variants, and reinfections on post-COVID-19 complications

In relation to carrying out possible observational studies investigating the effects of vaccination status, SARS-CoV-2 new variants, and reinfections on post-COVID-19 complications, we propose longitudinal and cross-sectional studies might be useful. The cross-sectional design of the study might be useful for monitoring and comparing multiple population samples observed and compared for the particular variables (e.g., population having post-COVID complications after SARS-CoV-2 reinfections vs. population having the post-COVID complications with no anamnesis of SARS-CoV-2 reinfection in order to investigate whether there are some particular symptoms occurring predominantly in the first of second condition). However, the selection of cross-sectional design is likely to be prone to multiple biases. First, there is a high probability of information bias stemming from comparing different population samples (between-subject design). Second, a cross-sectional study design is not suitable for describing the trends in the evolution of the particular variables, unless repeated measurements of the same populations' samples in ideally the same time periods are used (116). For that reason, longitudinal studies, which are used for repeated investigation of the particular variable within the same population sample, should be used for investigation of the duration of the particular post-COVID symptoms and their time-dependent intensity changes. The advantage of the possible use of longitudinal studies for the investigation of post-COVID complications might consist in its better signal-to-noise due to within-subject design compared to between-subject design in cross-sectional studies. We propose both retrospective and prospective longitudinal studies have advantages and disadvantages in their use (117, 118). A possible advantage of the retrospective longitudinal design of the study of post-COVID complications is that there are already existing databases of COVID-19-related complications of the previous pandemic waves in which the particular SARS-CoV-2 variant of concerns dominated and which might not be present anymore. Provided there exist databases related to post-COVID complications associated with each of the previous major pandemic waves, the evolution of post-COVID complications might be investigated. Furthermore, retrospective longitudinal studies would make it possible to analyze COVID-related data from the pre-vaccination era which would be of great importance for studying the link between post-COVID complications and vaccination status. On the other hand, prospective longitudinal studies dedicated to studying post-COVID complications would lead to data loss related to data from the pre-vaccination era as well as data related to post-COVID−19 complications attributable to the previous pandemic waves with their specific dominant SARS-CoV-2 variants. However, in contrast to retrospective studies, in prospective longitudinal studies, there would be a better signal-to-noise ratio due to a reduction of recall bias. It remains unknown how long the period for observation of post-COVID complications after SARS-CoV-2 infection or reinfection might last. However, health complications following SARS, which share some similarities with post-COVID complications (50), were documented to last for years (119). Provided that it would be the case of post-COVID complications, cohort studies with a long time observational period would be needed. Nevertheless, such investigation would be likely to be biased by the influence of other independent factors on the dynamics of post-COVID complications which are likely to come into play during long observational periods (118).

In relation to investigating whether there are some differences in effects of vaccination status, SARS-CoV-2 variants, and SARS-CoV-2 reinfections on responsiveness to various treatment modalities of participants suffering from post-COVID symptoms, we propose meta-analysis of clinical trials would be feasible. Furthermore, since the occurrence and severity of post-COVID complications may be dependent on demographic factors such as age (50, 120, 121), comorbidities (122), and gender (120), multiple clinical trials designed for a specific demographic population might reveal whether these kinds of demographic factors play a role in responsiveness to various treatment modalities for post-COVID complications.

## Other possible preventions against post-COVID manifestations

We believe that studying the dynamics of post-COVID-19 complications in relation to vaccination status, SARS-CoV-2 reinfections, and new SARS-CoV-2 variants is of considerable importance for a deeper understanding of the dynamics and mechanisms of post-COVID-19 complications and their prevention and treatment. Although vaccination status was documented to represent significant prevention against post-COVID complications (16, 17, 26), in immune-compromised people and/or in those whose vaccination status is not sufficient for prevention against post-COVID complications, other prevention strategies might be of great importance.

In spite of vaccination status, there were some risk factors associated with a higher risk of having post-vaccination SARS-CoV-2 infection despite being vaccinated. Those factors were the following: obesity (BMI > 30 kg/m2), highly deprived areas, and frailty (people over 60 years) (28). It remains unknown what exact causes are responsible for the link between these factors and higher risk of post-vaccination SARS-CoV-2 infection. From a statistical point of view, a higher prevalence of post-vaccination infection in the elderly population might be also linked with the higher prevalence of post-COVID complications in this particular population. This postulate might be at least partially supported by the documented link between age and the probability of developing post-COVID complications after SARS-CoV-2 infection (50, 54). Speaking about obesity, which is connected to chronic inflammatory conditions (123), obesity may represent a risk factor for the development of post-COVID conditions *via* maladaptive immune processes. For that reason, it is possible that other complex strategies apart from vaccination will be needed in obese people to minimize the risk of developing post-COVID manifestations. Regarding a positive correlation between highly deprived areas and higher risk of post-vaccination SARS-CoV-2 infection, during pandemic waves, highly deprived areas with a high density of population are likely to be exposed to high levels of viral loads which may represent a potential risk for increased probability of having some post-COVID manifestations (15). Furthermore, it is possible that in deprived areas, there is a lack of health services that would educate people about prevention strategies and treatments against COVID-19-related problems (28). Frailty was found to be associated with a higher probability of post-vaccination SARS-CoV-2 infection (28). This outcome might at least partially mimic the findings of other studies which reported a positive correlation between higher age and higher odds of developing post-COVID manifestations (53, 54, 124). This trend was attributed to immunosenescence and other age-dependent biological changes (28) which occur in elderly populations (125). For that reason, it can be assumed that elderly people might be also at a higher risk of developing post-COVID complications despite being vaccinated. For that reason, other prevention strategies should be developed to minimize the risk of long-term COVID-related disturbances.

### Physical exercise

Physical exercise was found to be associated with a significantly reduced risk of SARS-CoV-2 reinfections (126). Based on these outcomes, it is logical to assume that physical exercise might prevent post-COVID complications stemming from SARS-CoV- 2 reinfections as well. There are several possible supportive arguments for this hypothesis. First, physical exercise has the potential to improve the immune system (113, 127, 128). This notion may be of great importance for people with COVID-related immune pathologies after initial SARS-CoV-2 infection. Second, physical exercise has the potential to improve cardiovascular and pulmonary functions (129) whose malfunctioning occurs in post-COVID conditions (50, 59) and may considerably complicate successful recovery processes thereby representing risk conditions for worse outcomes related to SARS-CoV-2 reinfections. Third, physical exercise significantly reduces anxiety and depression (126) which are both associated with higher odds of developing post-COVID complications (69). Nevertheless, COVID-19 survivors may suffer from muscle atrophy (63) and reduced motor performance (130, 131). For that reason, physical exercise should be carefully regulated and individualized with regard to the type, intensity, and hardness of the particular exercise for the particular post-COVID patient as too intense and/or long physical exercise may induce pro-inflammatory processes (129) which would be likely to worsen the post-COVID condition.

### Treatment of co-morbidities

Apart from physical exercise, we propose that if there are some indications speaking in favor of the presence of some post-COVID complications following the initial infection, there should be treated with some appropriate therapy. Since neuropsychiatric manifestations, as well as pathologies emerging in various body systems, represent risk factors for developing post-COVID complications (59, 69, 79), elimination or at least maximal possible reduction of these conditions might be of great importance for reducing the risk of developing post-COVID manifestations after SARS-CoV-2 reinfection. To the best of our knowledge, the following possible types of treatments were shown to represent successful therapies for treating post-COVID−19 complications: pulmonary rehabilitation (132), psychotherapy (133), non-invasive transcranial neuro-modulation with direct current stimulation (134), pharmacological intervention (135) and immunotherapy (70, 71).

### Nutrition

Finally, nutrition may be another factor influencing the probability of developing post-COVID complications. Junk food such as chocolate was found to alter autonomic activity toward lower heart rate variability (HRV) (136) which is connected to a higher risk of malfunctioning of the autonomous nervous system (137). On the other hand, eating vegetables, such as carrots, was found to be associated with high HRV (136) which is the hallmark of the normal functioning of the autonomous nervous system (137). Since dysautonomia can manifest in post-COVID conditions (135), proper functioning of the autonomous nervous system is of great relevance when speaking about prevention strategies against post-COVID conditions. Nutrition can also modulate immune functions (138, 139) which might be of considerable importance for prevention strategies against post-COVID symptoms stemming from immune malfunctions.

Another supporting argument for the possible important role of nutrition in the prevention of post-COVID conditions might be found in the documented link between post-COVID complications and gut microbiota. In comparison to a healthy population, COVID-19 survivors were found to have altered microbiota (140). There was found a great abundance of opportunistic pathogens and a significant reduction of beneficial microbiota (36, 140). A positive association was documented between such kind of alteration of gut microbiota and worse COVID-19 outcomes. The following possible explanations for this phenomenon were proposed: First, an abundance of opportunistic pathogens and reduction of beneficial bacteria represent risk factors for developing immunopathologic conditions. Second, gut dysbiosis contributes to higher levels of baseline inflammation (141) and consequently leads to an increased probability of developing COVID-19-related complications associated with high-level baseline inflammation (33, 80). It has been found that some post-COVID manifestations positively correlate with the predominance of opportunistic pathogens and a low proportion of beneficial microbiota (35, 36). Since gut microbiota can be significantly modulated by nutrition (142), modulation of nutrition might play an important role in the prevention of post-COVID complications stemming from gut dysbiosis. This postulate might be supported by findings from the case study in which the participant suffering from post-COVID symptoms such as loss of appetite, anxiety, nausea, constipation, and nausea improved her condition after microbiota-targeted nutritional intervention (141).

Lastly, healthy and balanced nutrition also prevents obesity (28). Since obesity is associated with a higher risk of exacerbation of post-COVID complications, good nutrition habits might prevent post-COVID complications in indirect ways as well.

To sum it up, physical exercise, nutrition, and treatment of medical complications might represent successful prevention strategies against exacerbation of post-COVID complications. Physical exercise, nutrition, and treatment of medical complications might be of great importance, especially for people with positive anamnesis of SARS-CoV-2 infection, poor vaccination outcomes, or otherwise immunocompromised condition.

## Conclusion

This hypothesis article discussed the possible effects of vaccination status, SARS-CoV-2 reinfections, and new SARS-CoV-2 variants on post-COVID-19 complications and their mechanisms. Currently, vaccination seems to represent a significant step in preventing severe and long-lasting post-COVID-19 complications. Based on these outcomes, we propose that vaccination might eliminate some underlying mechanisms responsible for post-COVID complications and reduce the risk of developing post-COVID-19-related structural and functional abnormalities in tissues affected by COVID-19. The whole picture of health concerns related to infection and reinfection with different SARS-CoV-2 variants remains uninvestigated. Regarding the Omicron SARS-CoV-2 variant, which is the most recent variant of concern, our literature review found studies indicating lower as well as higher potential risk factors for exacerbation of post-COVID-19 complications following Omicron SARS-CoV-2 infection. Based on the current literature, the prevalence of post-COVID health problems seems to be lower in Omicron than it was for the previous dominating variants of concerns. We suggest that the mechanisms underlying potential post-COVID-19 complications might differ with regard to the particular SARS-CoV-2 variants. We believe that the severity and dynamics of post-COVID-19 complications and their underlying mechanisms should be studied in connection with SARS-CoV-2 reinfections. Finally, we hypothesize physical exercise, nutrition, and treatment of co-morbidities might reduce the risk of long-term post-COVID complications following initial SARS-CoV-2 infection as well as SARS-CoV-2 reinfection. The main source of limitations of our hypotheses consists in the current lack of literature related to the topic, especially to effects of SARS-CoV-2 reinfections and new SARS-CoV-2 variants on post-COVID complications and their underlying mechanisms. In spite of this limiting factor, we feel and hope our considerations are worth investigating for future research.

## Data availability statement

The original contributions presented in the study are included in the article/supplementary material, further inquiries can be directed to the corresponding author.

## Author contributions

MO wrote the preliminary and final manuscript versions. EK did proofreading and administration issues. All authors contributed to the article and approved the submitted version.

## Funding

This study was supported by Programme COOPERATIO of the Third Faculty of Medicine, Charles University.

## Conflict of interest

The authors declare that the research was conducted in the absence of any commercial or financial relationships that could be construed as a potential conflict of interest.

## Publisher's note

All claims expressed in this article are solely those of the authors and do not necessarily represent those of their affiliated organizations, or those of the publisher, the editors and the reviewers. Any product that may be evaluated in this article, or claim that may be made by its manufacturer, is not guaranteed or endorsed by the publisher.
